# Modern India and the Tale of Twin Nutrient Deficiency–Calcium and Vitamin D–Nutrition Trend Data 50 Years-Retrospect, Introspect, and Prospect

**DOI:** 10.3389/fendo.2019.00493

**Published:** 2019-08-09

**Authors:** Chittari Venkata Harinarayan, Harinarayan Akhila

**Affiliations:** ^1^Institute of Endocrinology, Diabetes, Thyroid and Osteoporosis Disorders, Sakra World Hospitals, Bengaluru, India; ^2^Department of Medicine & Endocrinology, Saveetha Institute of Medical and Technical Sciences University, Saveetha Medical College, Chennai, India; ^3^Information Services Group (ISG), Bangalore, India

**Keywords:** modern India, calcium and vitamin D deficiency, twin nutrient deficiency, tribal-rural-urban, nutrition time trends, RDA, RDI, strategies

## Abstract

Vitamin D and calcium are essential nutrients for bone health, to achieve peak bone mass and to preserve bone as age advances. A deficiency in these nutrients casts a long shadow in later life in the form of short/long latency diseases–rickets/osteomalacia/osteoporosis. There is scant review available about the trend of these nutrients in India. For over past half a century, the intake of dietary calcium, milk, milk products, and cereals has declined drastically in the background of upward revision of RDA/RDI in modern India. This is attributed to changing lifestyle, inadequate milk consumption across various socio-economic strata, and shift in dietary intake from cereals to rice and wheat. There is a clear rural–urban divide in consumption of milk, milk products and cereals, a change in dietary habits which magnify the calcium and vitamin D deficiency. Revisiting of RDA guidelines for calcium along with public health measures is required to tackle the morbidity arising due to the deficiency in these nutrients. Any measure to addresses this issue in isolation, without achieving the desired benefits, is a disservice to the population. Population based educational strategies, government measures, leveraging technology, adequate sun exposure and food fortification help in tackling the twin nutrient deficiencies in this diverse country.

## Introduction

Calcium and vitamin D are essential complementary nutrients for bone health. Withdrawal of a key nutrient (cause) and the occurrence of a disease (effect) results in nutritional deficiency disease. Tackling of short-latency diseases like beriberi, pellagra, rickets and scurvy were early successes of nutritional science. Osteoporosis is the index disease for calcium and is a long latency disease. To realize the full genetic potential of formation of skeletal mass, adequate calcium is required for growth. To maintain the skeletal mass attained, adequate calcium intake is required to offset the natural ongoing loss. It has been noted explicitly by National Academy of Sciences that a diet containing <50 mg calcium/100 Kcal results in osteoporosis (1–3). The recommended intake for maintenance is approximately three times this value (1, 2). Bone cannot be demineralized rapidly, even with zero calcium intakes. At slower rates of bone loss, it may take 30 years to produce severe depletion manifesting as fragility fractures. It is evident from studies of Chapuy et al. (4) and Dawson-Hughes (5) that older individuals supplemented with calcium and vitamin D were protected against non-vertebral fractures by 35–55% and age-related bone loss. In the treatment of age related bone loss and post-menopausal osteoporosis, vitamin D and calcium form an integral part of therapy. Without the mineral and Vitamin D, any anti-resorptive or anabolic agent will not have the desired effect of increasing the bone mineral density (BMD) (6). The secondary hyperparathyroidism (SHPT), an accompaniment of low dietary calcium intake (apart from maintaining normal serum calcium by leashing the bone), is also associated with high intracellular calcium ion concentrations (7).

Low calcium intake also results in various non-skeletal diseases. This increased intracellular concentration of calcium switches the adipocytes from lipolytic mode to liposynthetic mode and in some, hypertension. Fujita and Palmieri (8) coined “calcium paradox diseases,” common in old age as—arteriosclerosis, hypertension, diabetes mellitus, degenerative joint diseases, neurodegenerative diseases, and malignancy. The expression of calcium paradox diseases requires (a) Low dietary calcium intake (b) Increased intracellular concentration of calcium ion (impaired ability to pump calcium ions out of the cells), and (c) An environmental trigger—e.g., high salt intake for hypertension (8).

Rickets (or osteomalacia) is the index disease for vitamin D. Parfitt (9) defined 3 stages of “hypovitaminosis D osteopathy.” Stage 1—calcium malabsorption without histological changes (results in osteoporosis), stage 2—calcium malabsorption (osteoporosis) with wide osteoid seams (early osteomalacia) without clinical laboratory features indicative of osteomalacia, stage 3—severe vitamin D depletion with both clinical and histological rickets or osteomalacia. Hence, mild to moderate vitamin D deficiency is expressed solely as osteoporosis, not rickets or osteomalacia.

SHPT is an inevitable consequence of vitamin D deficiency referred to as hypovitaminosis D osteopathy stage-1 (HVO-1 or preosteomalacia). There is increase in osteoid surface. The osteoid thickness is usually <12.5 μm. There is no relationship between osteoid surface and osteoid thickness in normal subjects and patients with osteoporosis. With the progression of osteomalacia, the osteoid thickness is >12.5 μm and mineralization lag time is >100 days or infinity. In osteoporosis, the osteoid thickness is normal and the mineralization lag time is <100 days. In osteomalacia, the osteoid thickness correlates with osteoid and total bone surfaces (OS/BS) positively and negatively with adjusted mineral apposition rate (Aj.AR). The osteoid maturation time is normal in osteoporosis, but prolonged in osteomalacia. The osteoblast defect is in the matrix in osteoporosis. In osteomalacia, it is in the mineral (10).

In the tropical and subtropical regions of the world, nutritional rickets remains a public health problem in many developing countries (especially among infants, young children adolescents). Overcrowding, lack of sunlight exposure of lactating mothers and atmospheric pollution leading to vitamin D deficiency remain the commonest cause. There are recent evidences of dietary calcium deficiency-rickets developing in children with low calcium intakes. The dietary intake of calcium is ~1/3–1/2 the DRI for children in most of these developing countries (11).

The same fundamental physiological mechanisms operate for Rickets/Osteomalacia (short latency diseases) and osteoporosis (long latency disease), although at different levels. When there is vitamin D deficiency and dietary calcium depletion– rickets/osteomalacia occurs. Thus, calcium and vitamin D deficiency diseases constitute examples of short and long latency diseases (12). The dietary requirements of these nutrients are pegged to the prevention of the index diseases namely osteoporosis and rickets/osteomalacia. Rickets/osteomalacia is a qualitative defect of bone resulting from inadequate mineralization of the bone. Osteoporosis is a quantitative defect of bone when excess bone is lost (or inadequate bone formed).

### Nutrition Status in India: A Historical Perspective Since Independence

In the late nineteenth and early twentieth century, more than 80% of children living in United States and cities of Europe had growth deformities and features of rickets (13). During these centuries, the western world went through the industrial revolution, while India was affected by growing poverty and famine. When India became independent in 1947, even two square meals were not available for millions of households. The availability of cereals became an important goal to be achieved by the country. With this background, we now analyze the nutritional status of India since its independence.

## Nutrition Trends—India (1975–2017)

### Calcium

National Nutrition Monitoring Bureau (NNMB) provides data on nutritional status (based on households[HH]) of rural, tribal and urban populations in 10 out of 32 Indian states and union territories from 1975 to 2017 and their time trends over the period of time.

#### Rural Survey (Figures 1A–H)^1^

**Figure 1:**
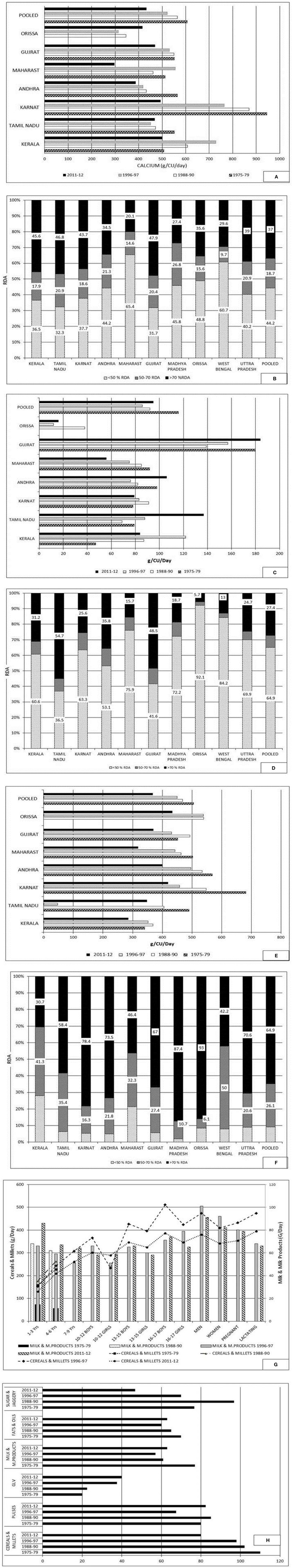
Rural survey. **(A)** Average consumption of calcium (g/CU/day): Time trends. Karnataka had recorded a fall of about 50% calcium intake from 946 (1975–79; RDA−400) to 493 (2011–12; RDA−600), Andhra Pradesh 565 (1975–79) to 388 (2011–12) and Maharashtra 512 (1975–79) to 297 (2011–12) g/CU/day. **(B)** Distribution percentage of households according to daily intake of calcium (RDA−600) as percent of RDA (Pooled). Only 37% of households (HH) had daily intake of calcium >70% of RDA and 44% <50% RDA (Pooled). The proportion of HH with calcium intake of <50% RDA was the highest in Maharashtra (65.4%) and lowest in Tamil Nadu and Gujarat (32% each). **(C)** Average consumption of milk and milk products (g/CU/Day): Time trends. **(D)** Distribution percentage of households according to daily intake of milk and milk products as percent of RDI (150) (Pooled). Only 27·4% of HH have daily intake of food rich in calcium of >70% RDI. Majority of HH (65%) have intake <50% of RDI. The percentage of HH with intake >70% of RDI are Tamil Nadu (54·7%) and Gujarat (48·5%). Major proportion of HH with intake <50 of RDI are Orissa (92%), West Bengal (84.2%), Maharashtra (76%), Madhya Pradesh (72%), Uttar Pradesh (70%), and Karnataka (63%). **(E)** Average consumption of cereals and millets (g/CU/Day; RDI−460): Time trends There has been a decline in intake of cereals and millets in past four decades with a median decrease—Karnataka (262 g), Andhra Pradesh (167 g), Maharashtra (183 g) and Tamil Nadu (142 g) and the lowest in Kerala (55 g). **(F)** Distribution percentage of households according to daily intake of cereals and millets as percent of RDI. Majority of HH (65%) had daily intake of cereals and millets >70% of RDI and 10% HH had intake of <50 of RDI (Pooled). **(G)** Average consumption of foods in different age groups–Time trends. **(H)** Average household intake of food stuffs as percentage of RDA: Time trends. Maharast-Maharashtra; Andhra–Andhra Pradesh; Karnat-Karnataka. Source: National Nutrition Monitoring Bureau—Technical report No 26–third report on rural survey^1^.

Trends over time of dietary calcium intake (g/CU/day) (grams per consumption unit per day) (pooled) shows a decline from 606 (1975–79) (RDA−400) to 433 in year 2011–2012 (RDA−600) (Figure 1A). The median intake was 331 g/CU/day. The percentage of HH with calcium intake of >70% of RDA ranged from 20 to 48% in different states (regions). The intake of milk and milk products (g/CU/day) (pooled) have declined from 116 (1975–79) to 95 (2011–12) (RDI−150) (Figures 1B,C). The intake of cereals and millets (g/CU/day) (pooled) declined from 505 (1975–1979) to 368 (2011–2012) (RDI−460) (Figures 1E,F). The median cereal and millet intake is 375 g/CU/day.

Across different age groups (infants, children, adults, pregnant, and lactating women) there has been a decline (from 1975–1979 to 2011–2012) in intake of calcium (Figure 1G), milk and milk products, and cereals (Figure 1H).

#### Tribal Survey (Figures 2A–H)^2^

**Figure 2:**
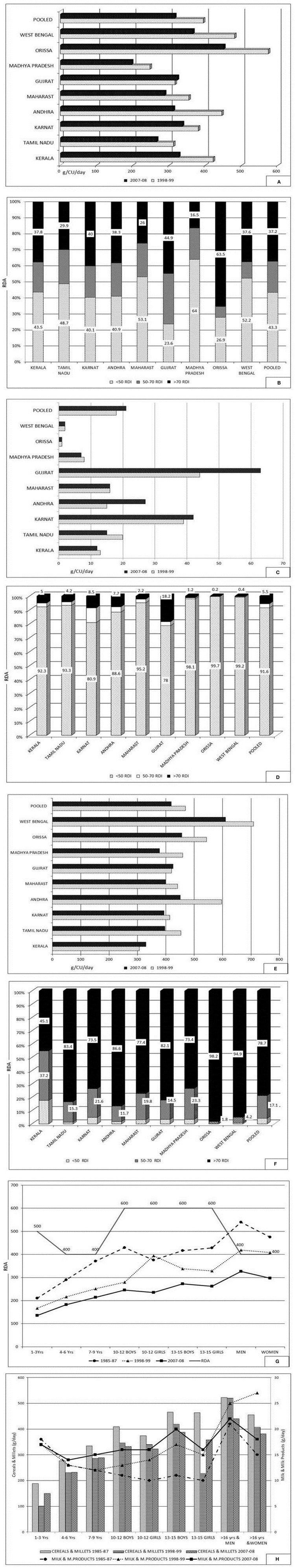
Tribal survey. **(A)** Average household intake of calcium (g/CU/day; RDA−400): Time trends. **(B)** Distribution percentage of households according to daily intake of calcium as percent of RDA. Only 37% of HH had dietary calcium intake of >70% of RDA and nearly 43% of HH had intake <50 of RDA (Pooled). The calcium intake was less than RDA (400) in all states, except in Orissa. In Orissa, 63.5% of HH consume >70% of RDA of calcium (450 CU/day), followed by Gujarat (45%), and Karnataka (40%). **(C)** Average household consumption of milk and milk products (g/CU/day; RDI−150): Time trends. **(D)** Distribution percentage of households according to daily intake of milk and milk products as percent of RDI. The intake was the lowest in Orissa (0.9 ml) and the highest in Gujarat (63 ml). In all states 80–100% (mean 91.6%) of the HH, the intake of milk and milk products was <50% of RDI. **(E)** Average household consumption of cereals and millets (g/CU/day): Time trends. The highest intake was in West Bengal (610) and lowest in Kerala (330) (year 2007–2008). **(F)** Distribution percentage of households according to daily intake of cereals and millets as percent of RDI (460). Majority of HH (79%) had intake of >70% of RDA and only 4% of HH had intake of <50 % of RDI (Pooled). **(G)** Average intake of calcium (g/CU/per day) in various age groups: Time trends. **(H)** Average intake of milk and milk products, and cereals and millets (g/CU/per day) in various age groups: Time trends. The intake of cereals and millets were >70% of RDI in majority of HH but still showed a decline compared to previous years (by 50 g). Source: National Nutrition Monitoring Bureau—Technical report No 25—Tribal survey^2^.

The trend over time of dietary calcium intake (g/CU/day) (pooled) over past two decades shows a decline from 394 (1998–1999) to 315 (2007–2008) (RDA 400) (Figure 2A). The median daily intake of calcium was 315 CU/day. The trends of intake of milk and milk products (g/CU/day) (pooled) shows a change from 18 (1998–1999) to 21 (2007–2008) (RDI 150) (Figure 2C).

The trends of intake of cereals and millets (g/CU/day) (pooled) declined marginally from 469 (1998–1999) to 419 (2007–2008) (RDI−460) (Figure 2E). Across different age groups, there has been low dietary calcium intake below the recommended RDA, and further decline compared to the years 1985–1987, 1998–1999, and 2007–2008 (Figure 2G). Similar trend was seen in intake of milk and milk products (Figure 2H).

#### Urban Survey (Figures 3A,B)^3^

The major portion of diet of the urban population was formed by cereals and millets (320 g/CU/day) which was lower than the RDA. Intake of milk and milk products were lower than RDI. About 67% of HH met the RDA requirement of calcium. Preschool children consuming <50% of RDA of calcium ranged from 2 to 93%.

**Figure 3:**
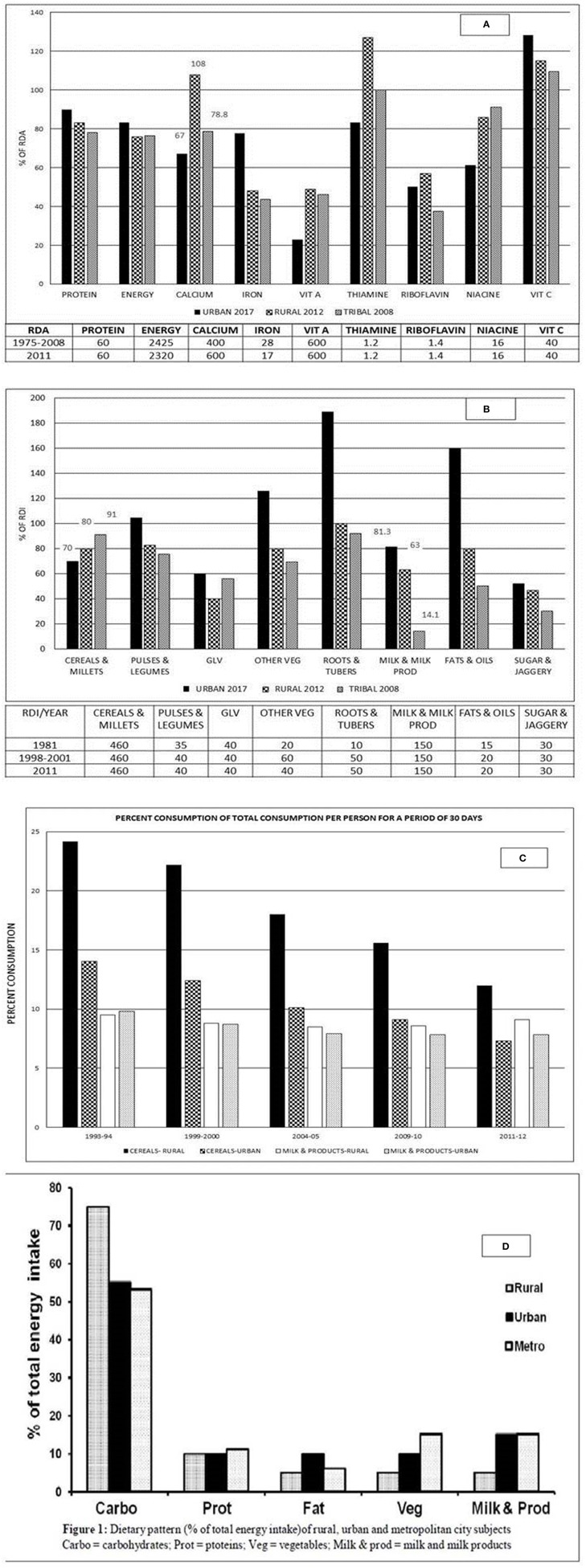
**(A)** Distribution of average house hold intake of nutrients as percentage of RDA in urban, rural and tribal surveys. **(B)** Distribution of average house hold consumption of food stuffs as percent of RDI in urban, rural, and tribal surveys. Vit A, vitamin A; Vit C, vitamin C; GLV, Green Leafy Vegetables; Other veg, other vegetables. **(C)** Percent distribution of total consumption per person for a period of 30 days urban, rural and tribal surveys—time trends. **(D)** Dietary pattern (percentage of total energy intake) of rural, urban, and metropolitan city subjects. Carbo, carbohydrates; Prot, proteins; Veg, vegetables; Milk and prod, milk and milk products. Source: **(A,B)** National Nutrition Monitoring Bureau—Technical report No 25,26,27—Tribal, rural and urban survey ^1^^,^^2^^,^^3^. **(C)** Socioeconomic statistics ministry of statistics 2017^8^. **(D)** Adapted from Harinarayan and Ramalakshmi (17). Sources of Calcium^8^ (mg/100 gm edible portion): Milk and milk products (120–210)—Buffalo's and cow's milk—curd; about 790–1,370 mg in cheese, paneer, Khoa, skimmed milk; Cereals and Legumes (200–340)-Ragi *(Eleusine coracana)*, whole Bengalgram *(Cicer arietinum)* and horsegram *(Macrotyloma uniflorum)*, rajmah *(Cicer Arietinum)*, and soya bean *(Glycine max)*; Green leafy vegetables (500–800)—Amaranth, cauliflower greens *(Brassica oleracea)*, curry leaves *(Murraya koenigii)*, knol-khol leaves *(Brassica oleracea var. gongylodes)*; Nuts and oil seeds-Coconut dry, almonds, hazel nuts, mustard *(Brassica)*, sunflower (130–490), gingelly *(Sesame)*, and cumin seeds *(Sesamum indicum)* (1,080–1,450). *Sources of Vitamin D:* Sunlight- UVB radiation, Cod liver oil, salmon fish, Mackerel, Sardines, Tuna, Egg yolk, Mushrooms (if exposed to sunlight or UV radiation).

From the NNMB data (1981–2011), there was a decline in dietary intake of calcium both in rural and tribal population in the past four and a half decades. The average HH intake of calcium as a percentage of RDA is 67% in urban (year 2017), 108% in rural (year 2012) and 78.8% in tribal (year 2008) subjects (Figure 3A)^1^^,^^2^^,^^3^. The distribution of HH consumption of milk and milk products were 81.3, 63, and 14% for urban, rural, and tribal population surveyed. The major source of calcium was through cereals and millets (70, 80, and 91% of urban, rural, and tribal population, respectively) (Figure 3B). While cereals and millets were the main source of calcium in rural and tribal HH, the urban HH received calcium from milk and milk products (Figure 3B) ^1^^,^^2^^,^^3^.

The National Sample Survey Organization (NSSO) conducts quinquennial surveys on monthly per capita food consumption at state and national level across all rural and urban households. The survey covers all states and union territories. It provides data on consumption expenditure of food and non-food items, and food security at the household level. The information collected during NSS 68th Round (2011–2012) is spread across the whole country, involving 83,935 urban and 119,378 rural HH (involving 7,469 rural and 5,268 urban blocks surveyed) (14). The major limitation of this data is that is collected through single interview, with a reference period of 30 days of consumption expenditure and provides no critical insight onto intrafamilial distribution of food consumption of individuals (15).

In both urban and rural areas, cereals form the main portion of diet followed by milk and fruits. Between 1987–1988 and 2009–2010 (NSSO 66th Round), the share of food in total consumer expenditure has fallen from 56 to 47% in urban and 64 to 54% in rural areas (16). The share of consumer expenditure of milk and milk products has declined from 9.5 to 7.8% in urban areas. But it is unchanged at 8.6% in rural areas. In the same period, there has been a fall in the share of consumer expenditure on cereals in rural (26–16%) and urban (15–9%) India, indicating a decline in cereal intake in the last three decades despite the ample availability.

Cereals contribute to 57% (rural) and 47% (urban) of energy intake. There was a progressive fall of cereal intake with rising monthly per capita expenditure (MPCE). The cereal consumption in rural subjects is 70% in the bottom 5% of the population, with a progressive fall to 42% in the top 5% of the population, as ranked by MPCE. In the urban population, it is 69% in bottom 5% of the population to 29% in top 5% of the population (NSSO 68th Round) (14). People in the lowest income group were consuming greater quantities of cereals despite spending a smaller portion of their income, because cereals were supplied through public distribution system (PDS). The rural population has changed the consumption pattern to rice and wheat as staple cereal. Coarse cereals (Ragi, maize, Jowar, and bajra) which are rich in micronutrients are not being consumed in substantial quantities. The decline in cereal consumption among middle and high income groups has been mainly due diet diversification in these groups consuming fast foods (Figure 3C)^4^.

#### Other Studies

Studies from South Indian subjects (rural, urban and metropolitan) (2015) showed significantly lower dietary calcium (*P* < 0.0001) in rural subjects (269 ± 2), urban (308 ± 2.3), and metropolitan subjects (526 ± 8) (mg/day; Mean ± SEM) (RDA−600) (17). The rural subjects had the highest intake of cereals and lowest intake of milk and milk products. The urban and metropolitan subjects had similar intake of cereals, milk and milk products (Figure 3D) (17). There are similar reports documented by other workers of low dietary calcium intake in adolescent (17) and post-menopausal women (18–20). Interestingly, a study of migrant workers in metropolitan cities had higher dietary calcium intake much above the RDA (21, 22).

### Vitamin D

**Figure 4:**
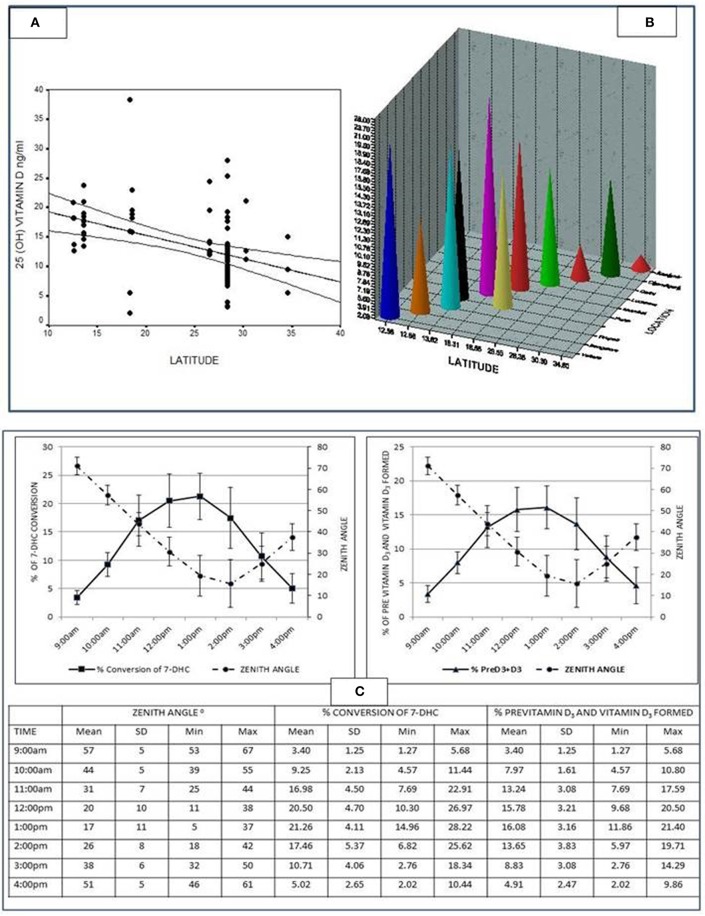
**(A)** Graph showing the inverse correlation between the 25(OH)D levels and latitude (*r* = −0·48; *p* < 0.0001) from various studies conducted in the country [Table 1 of (24)]. Adapted from Harinarayan et al. (24). **(B)** The 25(OH)D levels of various studies from India along with latitude and location from various studies conducted in the country [Table 1 of (24)]. Adapted from Harinarayan et al. (24). **(C)** Graph showing percent conversion of 7-Dehydrocholesterol (7-DHC) to previtamin D3 and photoproducts, and the percentage of previtamin D3 and vitamin D3 against time (for the study duration). The table below gives the individual values, mean ± SD minimum and maximum of the variables [Figure 2 of (25)]. Adapted from Harinarayan et al. (25).

The first ever documentation of low 25 Hydroxy vitamin D[25(OH)D] levels in India were on control population studied in a cohort of patients with primary hyperparathyroidism (23). Later other reports ensued. According to the study from India (24), about 85% of Indian population is suffering from various degrees of vitamin D deficiency. The 25(OH)D concentrations in subjects residing in north India are relatively lower compared to subjects in south India, with an inverse correlation with latitude (*r* = −0.48; *P* < 0.0001) (Figures 4A,B), probably because of equatorial closeness (zenith angle) (25). In a vitro study conducted at south India (Tirupati, Latitude 13.24^O^N) to study the synthesis of previtamin–D_3_ in an experimental model, it was shown that five times more previtamin-D_3_ and vitamin D_3_ formed at noon and 1 pm compared 9 to 10 a.m. (Figure 4C) (25).

### Modern India: Problem of Nutrition Inadequacy

India is a fastest growing economy which is self-sufficient in food production. The total population of India 960.5 million in 1995 went up to 1339.2 million in 2016 (26). As cities developed and enabled employment opportunities (urban migration, newer regions recorded as urban in census) and urban slums mushroomed, the percentage of rural population slumped from 73% (1995) to 65% (2016). With modernization and advent of machinery, agriculture became less labor intensive leading to fall in day-to-day in energy expenditure. Employment in agriculture fell from 61% (1995) to 43% (2016) (26). India's modernization includes sharp changes in lifestyle with long indoor working hours and changes in diet of erstwhile rural people with consumption of fast foods. With mechanization of agriculture less time is spent under the sun. Prolonged indoor working hours, modernization in culture and life-style leading to change in clothing habits, sun shy nature, and use of sunscreens with higher SPF are some of the factors causing hypovitaminosis D in India.

The production of cereals and milk has gone up from 85 (1995) to 123 (2016) and 68 (1995) to 164 (2016), respectively (Production indices 2004–2006 = 100). India is the largest milk producer in the world. About 80% of the milk production comes from small farmers (rural areas) (26, 27)^5^. Yet, the per capita monthly expenditure for consumption of milk and milk products in rural is Rs. 116 and urban area is Rs. 187 (27)^6^.

Thus, the new nutrition problem began to emerge in India- the problem of over-consumption and obesity. All this occurred in a single generation and constituted the dual burden—persistent undernutrition alongside emerging over nutrition—“The Nutrition Transition” (15). Hence, the reduction in undernutrition was matched by over nutrition and the normally nourished (60%) remained unchanged (15).

#### Changing Adequacy Ranges—RDA of Calcium and Vitamin D (Figures 5A,B)

**Figure 5:**
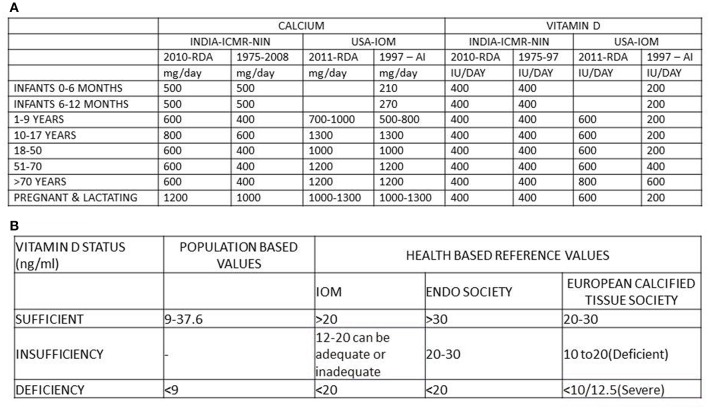
**(A)** RDA of calcium and vitamin D of India ICMR-NIN (27–29) and USA-IOM (31, 32)^9^. **(B)** Normal range of vitamin D from population based reference values, health based reference values (IOM & Endo. Soci). ICMR, Indian Council of Medical Research; NIN, National institute of nutrition; IOM, Institute of Medicine; Endo Soci, Endocrine society, USA; AI, adequate Intake; IU/day, International units/day; mg/day, milligram/day.

The RDA of calcium in India has been revised (28–30)^8^ but, is still lower than the revised RDA of USA and Canada (31). Similarly, the RDA of vitamin D of USA and Canada has been revised (31)^7^, while India's remains the same (28–30). Figure 5B summarizes vitamin D status according to different advisory bodies (29–31)^8^. The normal range of 25(OH)D concentrations to define vitamin D deficiency and sufficiency has undergone a change from population based reference range to health based reference range (33–36). The Endocrine Society guide lines are aimed at clinical care perspective. The IOM recommendations are directed at population health.

#### Interaction of Deficiencies of Calcium and Vitamin D

India is a diverse country with diverse cultural and dietary habits. The dressing and dietary habits vary between different regions and different socioeconomic classes. Indians come under the skin class category V (dark skin). With modernization and mechanization of lifestyle, the indoor working hours has increased, limiting sun exposure, and different dress codes in different regions of the country and population sects, increasing pollution, and increased usage of skin creams with SPF (Skin protection factor) >15 could account for the vitamin D deficiency.

The dietary calcium deficiency (with declining dietary calcium intake in India) (Figures 1–4) can lead on to secondary hyperparathyroidism (SHPT). This SHPT leads to increased conversion of 25(OH)D to 1,25–dihydroxyvitamin D, thereby reducing the serum 25OHD concentrations (37–41). The phosphaturic action of SHPT leads to low serum phosphorous and inadequate calcium phosphate ratio, resulting in rickets in children and osteomalacia in adults.

Studies from north India have shown vitamin D sufficiency in young adults and outdoor workers with a dietary calcium intake of 625 ± 273 mg/day and 405 ± 269 mg/day (21, 22, 42).

Studies from agricultural laborers in south India had 25OHD concentrations of ~23 ng/ml with dietary calcium 269 ± 2 (mg/day; Mean ± SEM) (24). The main source of dietary calcium in tribal, rural and semi urban areas are cereals and these contain Phytates, which retard the absorption of calcium (43, 44). Thus, the bioavailable calcium is far less than the RDA. With modernization leading to reduced cereal intake (Figure 3), and change of diet from coarse cereals to rice and wheat, the dietary calcium deficiency is further exaggerated. Intake of milk and milk products are near RDA only in urban populations (44). Thus, vitamin D deficiency leads to calcium malabsorption, which coupled with low dietary calcium leads to SHPT. Thus, urbanization, lifestyle changes, and change in dietary habits interact with one another to magnify the calcium and vitamin D deficiency. In addition, there are differences in food consumption pattern across regional (interstate) and socioeconomic classes (Figures 1–4) in India.

#### Impact of Twin Nutrient Deficiency

Calcium and phosphate are the main ions for bone stiffness. Calcitriol supplies these minerals by increasing the intestinal absorption of calcium and phosphate ingested daily. If there is no oral intake of these ions, calcitriol cannot do its job. Low dietary calcium supply and low vitamin D status causing calcium deprivation leads to SHPT. Though this SHPT maintains the serum calcium, sustained SHPT causes structural damage to bone and leads to hypophosphatemia (causing rickets/osteomalacia). In India, the dietary calcium plus vitamin D deficiency leads to rickets and/or osteomalacia in children and adolescents; in adults they lead to osteomalacia.

In vitamin D deficient states, the intestinal calcium absorption reduces to 10–15%. In vitamin D sufficient states, there is 30–80% calcium absorption (45).

#### Magnitude of the Problem

In the largest and longest study reported from India (1963–2005) (46), 52% of the population studied had nutritional bone disease. Osteomalacia (35.3%) and rickets (7.6%) caused by vitamin D deficiency due to inadequate sunlight exposure, dietary calcium deficiency (<300 mg/day) induced osteoporosis, vitamin D and calcium deficiency induced osteoporosis were the commonest disorders, besides fluoride interaction syndromes. Of the total population studies, about 40.6% had dietary calcium deficiency in critical years of growth. There are many reports of osteomalacia in adults from cross sectional studies (46–48).

Vitamin D deficiency can lead to low bone mass rickets in children and osteomalacia in adults. Vitamin D and calcium deficiency may lead to osteoporosis. In both the scenarios, there is increased risk of fractures. Compared to the Caucasians in the west where osteoporotic fractures occur mostly in females, in India, it occurs in both females and males, with most fractures among males. Also, fractures occur a decade or two earlier in Indians (49). Recent studies indicate that in Indian women, the BMD and peak bone mass are lower than that of Caucasians (50). Based on the clinical experience and available data (although exact numbers are lacking), an estimated 25 million may be affected with osteoporosis (51).

Hence, in the background of high prevalence of rickets, osteomalacia and subclinical vitamin D deficiency and decline in intake of calcium and low 25(OH)D levels the disease gets amplified, casting long shadows in later years of life. The peak bone mass is not reached in adulthood. There is a component of osteomalacia in osteoporosis in Indian context.

## The Way Forward: Strategies to Combat Nutrient Deficiencies

In the background of unsatisfactory levels of the combination of twin nutrient deficiency of calcium and vitamin D in India, any public health measure to address the issue in isolation may not yield the desired positive benefits for skeletal health. This problem requires to be addressed at various segments to realize the benefits, as soon as possible, by utilizing the available natural resources and fortification of food. Not only strategic initiatives, but also by leveraging technology as an enabler, both urban and rural population across India can be reached. In the west, calcium, and vitamin D are fortified using various strategies.

### Strategies That Can Be Implemented With Immediate Effect

#### Population Based Educational Strategies for Both the Nutrients

Population based educational strategies help to reach all segments of the society as early as possible until fortification can reach everyone and are also less expensive. The expertise of Indian nutrition scientist in increasing the availability of rice (which is not milled and polished) to lower socio-economic groups through the public distribution system (PDS) has resulted in natural death of Beriberi. Their dietary advise, to not solely rely on Jowar (sorghum-cause of pellagra) but instead, vary diet by including cereals and millets has led to disappearance of pellagra, which shows the success of educational strategies and implementation of remedial measures (52). These triumphs were seen early because of short latency diseases (beriberi and pellagra). But for calcium and vitamin D the results will be seen after a long latency.

One of the easiest approaches, for a population-based strategy, are educational programs describing the effects of this twin nutrient deficiency. This could involve (a) educating the children through their school curriculum and school teachers—The primary and secondary school curriculum can include this aspect not as a snippet, but as a complete module within the digital classroom sessions, every year, (b) updating medical college curricula pertaining to calcium and vitamin D status of the population, (c) education of expectant and breast feeding mothers in the antenatal clinics and during follow up in the post-partum period, (d) active participation of social service organizations, old age homes, (e) government mandate to involve the corporate sectors as a part of CSR (Corporate social responsibility), (f) print and electronic media to emphasize the importance of the twin nutrients in bone health. This will lead to increase in awareness among the public along with implementation of simple remedial methods to tackle the nutrient deficiency.

#### Calcium

Despite India being the largest producer of milk, the per capita consumption of milk and milk products is only 7.4, 15.3, 26.9, and 50.4% for very poor, poor, non-poor and rich, respectively (53). The majority of milk production is from the rural sector and they sell their produce for their livelihood.

India is combating the problem of milk adulteration/dilution which decreases the bioavailability of nutrients. Proper regulatory mechanisms should be enforced. Strategies to improve the awareness of diet diversification for lower socioeconomic population should be as follows: inclusion of balanced diet with cereal containing more calcium with less phytate content.

#### Vitamin D

FAO expert committee recommends an easy and physiologically relevant model of acquiring vitamin D through endogenous synthesis, which is sun exposure of skin (face and arms) for a minimum period of 30 min (without sun screen) to mid-day sun in most locations of the world, around the equator (between latitudes 42^0^ N and 42^0^ S) (54, 55). Studies on *in-vitro* model of vitamin D synthesis (24) which showed the maximal synthesis of Previtamin D and vitamin D (Figure 4) is validated by several other observational studies, from Chandigarh (41), Delhi (56–60), and Pune (61) suggesting the benefit of sunlight exposure to midday sun. Sunlight exposure (UV-B) has the potential to increase the serum 25OHD concentrations, but is limited by skin damage and skin cancer.

In schools, physical exercise should be made compulsory in the curriculum. Students should be encouraged to expose themselves in sun as much as possible in lunch hour—“sunshine hour” (55). The efforts should be aimed at educational programs among physicians and general public at large, updating medical curriculum regarding the benefits of vitamin D and its effects on deficiency (62–66). Bioaddition of vitamin D include increasing the vitamin D content of eggs, farm fish, livestock (meat), UV exposure of mushrooms and yeast (then used for bread) are some of the methods (67).

#### Targeted Supplementation

Supplementation of calcium and vitamin D in antenatal clinics and post-partum mothers would not only benefit the bone health of mother and child, but also reduce pre-eclampsia and caesarian sections (68). There are reports of inverse association of serum 25(OH)D levels and cesarean sections (68). Low serum 25(OH)D levels are also associated with increased primary C-section, uterine atony and post-partum hemorrhage (68, 69).Similar approaches should be considered in government run shelters, old age homes, and residential communities for retired staff and the infirm.

### Long Term Measures

Nutritionists in India have shown success in implementation of salt iodization program. Hence, fortification of milk, cereals and staple foods with calcium and vitamin D should be considered. There is a decline in intake of cereals and shift to rice and wheat in Indian population. Hence, these grains, their flour and related products should be the targets for fortification, apart from milk and oil, to cover the entire population. It could be mass fortification, targeted or market driven fortification. Presently Milk, Oils-edible oils, rice bran oils, sunflower oil, Vanaspathi, wheat flour are fortified with vitamin D—mandatory in India. Fortified foods include—milk and milk variants, soya milk, juices (minute maid) and biscuits with calcium and vitamin D (70).

#### Targeted Fortification

People in lower socioeconomic strata procure their cereals and staple grains from the PDS. It will be an imperative to maintain the fortified standards of calcium and vitamin D for this segment.

Food supplied to schools for mid-day meal schemes should procure fortified rice, other cereals and their products for preparation of their meals. Similarly, food supplied by ICDS (Integrated Child Development Scheme) should use fortified grains. In addition, supply of fortified milk to children as a part of ICDS and mid-day meal schemes could also be considered. Non- profit organizations like ISKCON (International Society for Krishna Consciousness), Akshaya Patra who distribute mid-day meal to school children can get involved in using fortified food grains for meal preparations, distribution of fortified, ready-to-eat food to school children during their evening snacks like sattu (62–65).

#### Mass Fortification

Another strategy to combat twin nutrient deficiency can be through mass fortification which would involve recurrent costs in production, marketing and education costs, and other program specific costs like monitoring and surveillance etc. The benefit of fortification would be seen over a period of time (time to achieve complete fortification and because of long latency disease).

As both calcium and vitamin D are essential complementary nutrients for maintaining skeletal health which are deficient in Indian population, there should be a government mandate (legislation) to fortify calcium and vitamin D.

Fortification of milk is a time tested and a validated method (does not require to be validated by any further studies in any groups of population in any other region) which might address the bulk of calcium and vitamin D deficiencies, but would benefit only 35% of the population (urban). The consumption of milk (Kg/capita/annum) (2009–2010) in rural area is 51.7 and in urban area is 71.6. The share in monthly per capita food expenditure is 14.9 and 18.4% in rural and urban areas, respectively ^5^^,^^6^^,^^7^. Apart from fortification of milk and oil to cover all segments of population, other food items that can be fortified includes rice, wheat, cereals and their products in addition to sugar and its variants (62–65). In India, the staple foods are being fortified with vitamin A and essential minerals. It is an easy approach to fortify them with calcium and vitamin D along with other nutrients in the same process.

#### Market Driven Fortification

Ready-to-eat foods such as canned fruit juices, health drinks, health/granola bars, biscuits, toffees, sweets etc. can be fortified with calcium and vitamin D. This helps the corporate companies to expand their market share by targeting growing children, pregnant and lactating mothers, the health conscious population, and people recovering from chronic illness.

#### Pros and Cons of Mandatory vs. Voluntary Fortification

Food fortification has traditionally been implemented using a mandatory approach. However, voluntary fortification is also effective in mature markets. The demand is mostly seen for branded food products with information of fortification included in the packaging. Launching fortification through voluntary approaches attracts goodwill from the local authorities and the freedom to impose own prices. In a drive to increase the market share through robust communication strategies, there are huge investments required. The need to spread the awareness of the health benefits of fortified food lies with the enterprise that opt for voluntary food fortification leading to lower cost-benefit ratio. The limitations are the inability to sustain the cost of producing new fortified products, huge investments in communication strategies to spread awareness of the benefits from fortified food. Also, as many staple foods are sold in loose and unbranded form, it is difficult to incorporate fortification logos on a product without a package. On the other hand, mandatory food fortification allows implementing new standards with centrally regulated and subsidized prices with a higher cost-benefit ratio as the government mandates the fortification. It is cost effective with an immediate public health impact. However, the supply chain needs to be supportive for a mandatory food fortification and the local producers should be ready to enforce the fortification regulations.

### Role of Technology in Tackling Calcium and Vitamin D Deficiency

Technology is an enabler for the implementation of strategies to improve public health nutrition. In this aspect, the use of internet and mobile phones for communicating health benefits has seen a positive response (71). Hence, the ability to provide personalization is a major focus area for healthcare technology providers. Biosensors in smartphones are being used to assess the health outcomes and to re-calibrate the nutritive supplementation for the user (72). Gamification (virtual games using points as motivator) is also a proven methodology to improve awareness of daily nutrition intake among children (73).

In India, the mobile phone penetration in mid-2017 is 57%^9^ and the total mobile phone customer base reached 1.17 billions by the end of the year^10^. The reach of mobile internet is estimated to be 60.8% and 39.1% in urban and rural India by June 2018^11^. The internet penetration in urban and rural India is 64.84% and 20.26% as on December 2017. It is estimated only 30% of total internet users are female, indicating a huge gender gap in digital literacy^12^.

While mobile apps are one of the easiest methods of reaching to a wide-spread population segment, other approaches include advertisements in print and social media, health-based interactive videos, and on-call nutritionists. Use of emerging technologies, such as analytics, sensors and artificial intelligence to assess the calcium and vitamin D deficiency and to predict the duration of recovery helps in improving public health nutrition^13^. In the regions where internet is impenetrable, other approaches such as on-call nutritionists, call centers to answer nutrition-specific queries should be leveraged as a strategy to increase awareness of calcium and vitamin D deficiency and the remedial measures.

Bupa Healthcare has implemented over-the-phone personalized nutrition coaching for their members. This has helped patients to eat healthier and live a healthy lifestyle^14^^,^^15^. HealthPhone, a project of The Mother and Child Health and Education Trust (MCHET), a non-profit charitable trust, founded to establish communications technologies that will provide health and nutrition information to and from rural low-income communities, provides a video reference library and guide to better health and nutrition practices. This trust has distributed mobile phones with access to this video library for families and communities, including the illiterate, to view the videos in their native language, distributed on mobile phones^16^ (74).

#### Calcium

Apart from milk and milk products, there are calcium-rich foods which are available across India. However, the adoption of calcium-rich foods in addition to milk is very low. Some reasons that could be attributed to this cause are lack of awareness, non-availability of specific food items in a region, and lack of culinary expertise to consume these foods.

Through different means of communication to the urban and rural populations, awareness can be increased by suggesting measures which are localized for the focus region. Target programs for a specific region can be undertaken to include dietary calcium intake through localized culinary delicacies specific to the region or the cultural background of the target segment.

#### Vitamin D

Studies have proven the effectiveness of mobile apps for assessing vitamin D levels and suggesting remediation methods (75). The assessment leverages a smart phone accessory and a test strip which in integration with the smartphone app provides the test results (76). However, these apps do not provide the nitty-gritties of the easiest remediation method—sun exposure, and none of these apps are available in India.

It is an imperative when launching an app to indicate the optimal duration of sun exposure and the vitamin D acquired during the exposure. Dminder app provides this, by leveraging the GPS location of the phone, weather conditions and the solar positioning at the location.

#### The Integrated Approach

The combination of increase in awareness, adoption of localized approach and the support of government for ethical technological use is an imperative for combating calcium and vitamin D deficiency. While mobile apps or on-call services can be provided to understand nutritive values of food or food products available through various channels of sale, however, localized programs strategized to target a specific micro region will need a regional expertise.

#### Governmental Measures

1. Revision of RDA of calcium
2. Price regulation of calcium and vitamin D supplements under price control act (to be made a mandate by legislation)—to be affordable to even lower socio economic segments of the society–so that supplementation is affordable and feasible
3. Supplementation of calcium and vitamin D to all pregnant and lactating mothers in primary health centers
4. Regulate the calcium and vitamin D content in multivitamins
5. Benefits to corporate companies who are involved in mandatory food fortification, in the form of additional tax benefits for mandatory food fortification initiatives, rebates as a part of corporate social responsibility (CSR), availability of fortified food in corporate cafeterias leading to better employee well-being, thereby increasing productivity
6. Support to research groups to study the impact of vitamin supplementation and fortification programs in all parts of the country. Support in the form of approvals for research projects and provide financial assistance for a detailed study in this sector along with assessing the outcomes. Also, provide support to implement the findings of the project
7. Policies for implementing ethical use of technology to optimize channels for enhancing public health nutrition.

## Conclusion

There is a widespread dietary calcium and vitamin D deficiency in India. For over half a century, the intake of dietary calcium, milk, milk products, and cereals has declined drastically. There is a clear rural–urban divide in consumption of milk, milk products and cereals. The deficiency of these nutrients manifests as rickets/osteomalacia and/or osteopenia/osteoporosis, which is realized at much later age. Stunting and some of the calcium paradox diseases could be attributed to the deficiency of these nutrients. Fortification of food with only vitamin D in India in the background of dietary calcium deficiency is doing a disservice to the population/society. There is an urgent need to address these issues by the scientific societies and the Government of India through strategic measures or through food fortification of calcium and vitamin D. Until these programs see the light of the day, it is prudent on our part to obtain vitamin D by most inexpensive way, which is by exposure to sunlight and meeting the RDA of calcium in our diet or supplementation to mineralize the osteoid in the most easiest way (66, 77, 78). It may be a golden opportunity for school going children to expose themselves in sunlight and get adequate calcium in their mid-day meals to achieve the peak bone mass (77).

## Search Strategy and Selection Criteria

References for this review were identified through searches of PubMed and Google scholar for articles published from January 1971 to December 2018, by using the terms “dietary Calcium Intake India,” “Vitamin D status India,” “Sunlight Vitamin D India,” “Fortification of Milk India,” “RDA India, IOM, USA, and Canada,” “Nutrition apps,” “technology nutrition,” “mobile apps vitamin D calcium,” “mobile internet India.” Article resulting from these searches and relevant references cited in these articles were reviewed. Studies which contained both dietary calcium intake and vitamin D status were taken for major consideration, except where the dietary calcium alone was given in recent studies were taken. For data on dietary calcium intake from 1970 till date, time trends were sourced from NIN-ICMR (national Institutes of Nutrition and Indian council of Medical Research) and NFI (nutrition foundation of India) websites. Data was also sourced from National Sample Survey Office (NSSO)—Ministry of statistics and program implementation (MOSPI)-India, National Family Health Survey (NFHS) by International Institute of population sciences (IIPS), National dairy development board (NDDB) for data on milk consumption of India and FAO web site—Food and agricultural Organization of the United nations.

## Author Contributions

CH conceptualized study design and did relevant literature search, data collection, analysis, interpretation, preparation of figures, writing of the manuscript. HA was practically and intellectually involved in the section on Role of Technology in Tackling Calcium and Vitamin D Deficiency, searched and reviewed relevant literature, participated in data collection, analysis and interpretation. development of figures, writing, and language correction of the article.

### Conflict of Interest Statement

The authors declare that the research was conducted in the absence of any commercial or financial relationships that could be construed as a potential conflict of interest.

## Footnotes

^1^http://nnmbindia.org/1_NNMB_Third_Repeat_Rural_Survey___Technicl_Report_26.pdf (accessed January 1, 2019).

^2^http://nnmbindia.org/NNMBTribalReport.pdf (accessed January 1, 2019).

^3^http://ninindia.org/NNMB%20Urban%20Nutrition%20survey%20%20report-Final%2025-09-2017.pdf (accessed January 1, 2019).

^4^http://mospi.nic.in/sites/default/files/publication_reports/SelectedSocio-EconomicStatisticsIndia2017_27oct17.pdf (accessed December 31, 2018).

^5^https://www.worldatlas.com/articles/top-cows-milk-producing-countries-in-the-world.html (accessed December 29, 2018).

^6^https://www.nddb.coop/sites/default/files/pdfs/merged_document9-10.pdf (accessed December 29, 2018).

^7^https://www.ncbi.nlm.nih.gov/books/NBK56070/ (accessed January 1, 2019).

^8^http://ninindia.org/DietaryGuidelinesforNINwebsite.pdf (accessed January 1, 2019).

^9^https://thenextweb.com/contributors/2017/07/13/india-overtakes-usa-become-facebooks-top-country/ (accessed January 4, 2019).

^10^https://economictimes.indiatimes.com/tech/internet/internet-users-in-india-expected-to-reach-500-million-by-june-iamai/articleshow/63000198.cms (accessed January 4, 2019).

^11^https://www.reuters.com/article/india-telecoms-users/table-indias-mobile-phone-users-rise-4-97-mln-to-1-17-bln-in-dec-idUSL4N1Q61KZ (accessed January 4, 2019).

^12^https://yourstory.com/2018/03/mobile-internet-users-india-reach-478-million-june-2018-iamai-report/ (accessed January 4, 2019).

^13^http://www.ourbupa.co.uk/Pages/Health-coach-team.aspx (accessed May 23, 2019).

^14^https://www.bupa.com.au/dietitianhealthcoaching (accessed May 23, 2019).

^15^http://www.healthphone.org/ (accessed May 23, 2019).

^16^http://www.healthphone.org/about.htm (accessed May 23, 2019).

## References

[1] Forbes RM Weingartner KE Parker HM Bell RR Erdman JW Jr. Bioavailability to rats of zinc, magnesium and calcium in casein-, egg and soy protein–containing diets. J Nutr. (1979) 109:1652–60. 10.1093/jn/109.9.1652 573313

[2] Guéguen L Pointillart A. The bioavailability of dietary calcium. J Am Coll Nutr. (2000) 19(Suppl. 2):119S−36. 10.1080/07315724.2000.10718083 10759138

[3] Institute of medicine (US) committee to review dietary reference intakes for vitamin D and calcium In: Ross AC, Taylor CL, Yaktine AL, Del Valle HB, editors. Dietary Reference Intakes for Calcium and Vitamin D. Washington, DC: National Academies Press (US) (2011). 21796828

[4] Chapuy MC Arlot ME Duboeuf F Brun J Crouzet B Arnaud S. Vitamin D3 and calcium to prevent hip fractures in elderly women. N Engl J Med. (1992) 327:1637–42. 10.1056/NEJM199212033272305 1331788

[5] Dawson-Hughes B Harris SS Krall EA Dallal GE Effect of calcium and vitamin D supplementation on bone density in men and women 65 years of age or older. N Engl J Med. (1997) 337:670–6. 10.1056/NEJM199709043371003 9278463

[6] Cummings SR San Martin J McClung MR Siris ES Eastell R Reid IR. Denosumab for prevention of fractures in postmenopausal women with osteoporosis. N Engl J Med. (2009) 361:756–65. 10.1056/NEJMoa0809493 19671655

[7] Palmieri GMA Nutting DF Bhattacharya SK Bertorini TE Williams JC. Parathyroid ablation in dystrophic hamsters. J Clin Invest. (1981) 68:646–54. 10.1172/JCI110299 7276164 PMC370845

[8] Fujita T Palmieri GMA. Calcium paradox disease: calcium deficiency prompting secondary hyperparathyroidism and cellular calcium overload. J Bone Miner Metab. (2000) 18:109–25. 10.1007/s007740050101 10783844

[9] Parfitt AM Osteomalacia and related disorders. In: Avioli LV, Krane SM, editors. Metabolic Bone Disease and Clinically Related Disorders. 2nd ed. Philadelphia, PA: WB Saunders (1990). p. 329–96.

[10] Bhan A Qiu S Rao SD. Bone histomorphometry in the evaluation of osteomalacia. Bone Rep. (2018) 8:125–34. 10.1016/j.bonr.2018.03.005 29955631 PMC6020114

[11] Pettifor JM. Calcium and vitamin D metabolism in children in developing countries. Ann Nutr Metab. (2014) 64(Suppl. 2):15–22. 10.1159/000365124 25341870

[12] Heaney RP. Long-latency deficiency disease: insights from calcium and vitamin D. Am J Clin Nutr. (2003) 78:912–9. 10.1093/ajcn/78.5.912 14594776

[13] Holick MF Vitamin D is not as toxic as was once thought: a historical and an up-to-date perspective. Mayo Clin Proc. (2015) 90:561–4. 10.1016/j.mayocp.2015.03.015 25939933

[14] NSS, – 68^th^ Round,. Available online at: http://mospi.nic.in/sites/default/files/publication_reports/nss_report_560_19dec14.pdf (accessed December 29, 2018).

[15] Ramachandran P The Double Burden of Malnutrition in India. Available online at: http://www.fao.org/tempref/docrep/fao/009/a0442e/a0442e01.pdf (accessed December 29, 2018).

[16] NSS – 66 th Round. Available online at: https://data.gov.in/catalog/household-consumer-expenditure-national-sample-survey (accessed December 29, 2018).

[17] Harinarayan CV Ramalakshmi T Patterns of dietary calcium intake in south Indian rural, urban and metropolitan city subjects. J Clin Sci Res. (2015) 4:143–8. 10.15380/2277-5706.JCSR.15.003

[18] Patil S Joglekar C Desai M Yadav A Sonawane S Chavan R Nutritional status and psychological impairment in rural adolescent girls: pilot data from “KOKAN” region of Western India. Front Public Health. (2018) 21:160 10.3389/fpubh.2018.00160 PMC6021506 29977886

[19] Raj JP Oommen AM Paul TV. Dietary calcium intake and physical activity levels among urban South Indian postmenopausal women. J Family Med Prim Care. (2015) 4:461–4. 10.4103/2249-4863.161355 26288793 PMC4535115

[20] Harinarayan CV. Prevalence of vitamin D insufficiency in postmenopausal south Indian women. Osteoporos Int. (2005) 16:397–402. 10.1007/s00198-004-1703-5 15776219

[21] Goswami R Saha S Sreenivas V Singh N Lakshmy R. Vitamin D-binding protein, vitamin D status and serum bioavailable 25(OH)D of young Asian Indian males working in outdoor and indoor environments. J Bone Miner Metab. (2017) 35:177–84. 10.1007/s00774-016-0739-x 26832389

[22] Harinarayan CV Letter to the editor on “Vitamin D-binding protein, vitamin D status and serum bioavailable 25(OH)D of young Asian Indian males working in outdoor and indoor environments”. J Bone Miner Metab. (2017) 35:243–4. 10.1007/s00774-016-0776-5 27623789

[23] Harinarayan CV Gupta N Kochupillai N. Vitamin D status in primary hyperparathyroidism in India. Clin Endocrinol. (1995) 43:351–8. 10.1111/j.1365-2265.1995.tb02043.x 7586606

[24] Harinarayan CV Ramalakshmi T Prasad UV Sudhakar D. Vitamin D status in Andhra Pradesh: a population-based study. Indian J Med Res. (2008) 127:211–8. 18497434

[25] Harinarayan CV Holick MF Prasad UV Vani PS Himabindu G. Vitamin D status and sun exposure in India. Dermatoendocrinol. (2013) 5:130–41. 10.4161/derm.23873 24494046 PMC3897581

[26] FAOSTAT Available online at: http://www.fao.org/faostat/en/- (accessed December 29, 2018).

[27] Milk Consumption Data – India. Available online at: https://www.nddb.coop/information/stats/percapitacomsp (accessed December 29, 2018).

[28] Expert Group of ICMR Recommended Dietary Intakes for Indians. ICMR (1981). Available online at: https://www.icmr.nic.in/content/nutrient-requirements-recommended-dietary-allowances-indians-1990-reprinted-2008-2nd-edition (accessed December 28, 2018).

[29] Report of the Expert Group of the Indian Council of Medical Research Nutrient Requirements and Recommended Dietary Allowances for Indians. New Delhi: Indian Council of Medical Research (2010). Available online at: https://www.icmr.nic.in/content/nutrient-requirements-recommended-dietary-allowances-indians (accessed December 28, 2018).

[30] Revised, RDA for Indians,. Available online at: http://nutritionfoundationofindia.res.in/PPT-2011/Seven17-18teen/Dr-B-Sesikeran.pdf (accessed December 29, 2018).

[31] Ross AC Manson JE Abrams SA Aloia JF Brannon PM Clinton SK. The 2011 report on dietary reference intakes for calcium and vitamin D from the institute of medicine: what clinicians need to know. J Clin Endocrinol Metab. (2011) 96:53–58. 10.1016/j.jada.2011.01.004 21118827 PMC3046611

[32] Lips P Cashman KD Lamberg-Allardt C Bischoff-Ferrari HA Obermayer-Pietsch BR Bianchi M. MANAGEMENT OF ENDOCRINE DISEASE: Current vitamin D status in European and Middle East countries and strategies to prevent vitamin D deficiency; a position statement of the European Calcified Tissue Society. Eur J Endocrinol. (2019). 10.1530/EJE-18-0736. [Epub ahead of print]. 30721133

[33] Lips P. Vitamin D deficiency and secondary hyperparathyroidism in the elderly: consequences for bone loss and fractures and therapeutic implications. Endocr Rev. (2001) 22:477–501. 10.1210/edrv.22.4.0437 11493580

[34] Holick MF Binkley NC Bischoff-Ferrari HA Gordon CM Hanley DA Heaney RP. Evaluation, treatment, and prevention of vitamin D deficiency: an Endocrine Society clinical practice guideline. J Clin Endocrinol Metab. (2011) 96:1911–30. 10.1210/jc.2011-0385 21646368

[35] Rosen CJ Abrams SA Aloia JF Brannon PM Clinton SK Durazo-Arvizu RA. IOM committee members respond to Endocrine Society vitamin D guideline. J Clin Endocrinol Metab. (2012) 97:1146–52. 10.1210/jc.2011-2218 22442278 PMC5393439

[36] Harinarayan CV What's in a name −25(OH)D or 25(OH)D3? Natl Med J India. (2004) 17:114; author reply 114–5. Available online at: http://archive.nmji.in/Correspondence/What%92s_in_a_name_25(OH)D.htm 15141611

[37] Pfeifer M Begerow B Minne HW Abrams C Nachtigall D Hansen C. Effects of a short-term vitamin D and calcium supplementation on body sway and secondary hyperparathyroidism in elderly women. J Bone Miner Res. (2000) 15:1113–8. 10.1359/jbmr.2000.15.6.1113 10841179

[38] Mellanby E An experimental investigation on rickets. Lancet. (1919) 1: 407–12. 10.1016/S0140-6736(01)25465-8 794773

[39] Sly MR van der Walt WH Du Bruyn D Pettifor JM Marie PJ. Exacerbation of rickets and osteomalacia by maize: a study of bone histomorphometry and composition in young baboons. Calcif Tissue Int. (1984) 36:370–9. 10.1007/BF02405348 6435836

[40] Heaney RP Vitamin D depletion and effective calcium absorption. A letter to the editor. J Bone Min Res. (2003) 18:1342 10.1359/jbmr.2003.18.7.1342 12854846

[41] Heaney RP Dowell MS Hale CA Bendich A. Calcium absorption varies within the reference range for serum 25-hydroxyvitamin D. J Am Coll Nutr. (2003) 22:142–6. 10.1080/07315724.2003.10719287 12672710

[42] Ramakrishnan S Bhansali A Bhadada SK Sharma R Walia R Ravikiran M. Vitamin D status and its seasonal variability in healthy young adults in an Asian Indian urban population. Endocr Pract. (2011) 17:185–91. 10.4158/EP10155.OR 20841308

[43] Harinarayan CV Ramalakshmi T Prasad UV Sudhakar D Srinivasarao PV Sarma KV. High prevalence of low dietary calcium, high phytate consumption, and vitamin D deficiency in healthy south Indians. Am J Clin Nutr. (2007) 85:1062–7. 10.1093/ajcn/85.4.1062 17413106

[44] Harinarayan CV Ramalakshmi T Venkataprasad U. High prevalence of low dietary calcium and low vitamin D status in healthy south Indians. Asia Pac J Clin Nutr. (2004) 13:359–64. 15563441

[45] Misra M Pacaud D Petryk A Collett-Solberg PF Kappy M Drug and therapeutics committee of the lawson wilkins pediatric Endocrine Society. Vitamin D deficiency in children and its management: review of current knowledge and recommendations. Pediatrics. (2008) 122:398–417. 10.1542/peds.2007-1894 18676559

[46] S Teotia SP Teotia M. Nutritional bone disease in Indian population. Indian J. Med Res. (2008) 127:219–8. 18497435

[47] Rajeswari J Balasubramanian K Bhatia V Sharma VP Agarwal AK. Aetiology and clinical profile of osteomalacia in adolescent girls in northern India. Natl Med J India. (2003) 16:139–42. Available online at: http://archive.nmji.in/archives/volume%2016-3%20may%20june%202003/original%20article/Aetiology_and_clinical.htm 12929856

[48] Sachan A Gupta R Das V Agarwal A Awasthi PK Bhatia V. High prevalence of vitamin D deficiency among pregnant women and their newborns in northern India. Am J Clin Nutr. (2005) 81:1060–4. 10.1093/ajcn/81.5.1060 15883429

[49] Gupta A. Osteoporosis in India–the nutritional hypothesis. Natl Med J India. (1996) 9:268–74. 9111786

[50] Shatrugna V Kulkarni B Kumar PA Rani KU Balakrishna N. Bone status of Indian women from a low-income group and its relationship to the nutritional status. Osteoporos Int. (2005) 16:1827–35. 10.1007/s00198-005-1933-1 15959616

[51] Malthotra N Mithal A Osteoporosis in Indians. Indian J Med Res. (2008) 127:263–8. 18497441

[52] Gopalan C. The changing nutrition scenario. Indian J Med Res. (2013) 138:392–7. Available online at: http://www.ijmr.org.in/text.asp?2013/138/3/392/119380 24135189 PMC3818608

[53] Kumar A Joshi PK Kumar P Parappurathu S Trends in the consumption of milk and milk products in India: implications for self-sufficiency in milk production. Food Sec. (2014) 6:719–26. 10.1007/s12571-014-0376-y

[54] Report of the Joint FAO/WHO Expert Consultation on Vitamin Mineral Requirement in Human Nutrition: Bangkok 1998, 2 ed. Rome: FAO (2004). Available online at: http://www.fao.org/3/a-y2809e.pdf (accessed January 1, 2019).

[55] Srinivasa PM Harinarayan CV Vitamin D deficiency in India: fortify or let the sun shine in? JCSR. (2015) 4:220–6. 10.15380/2277-5706.JCSR.15.024

[56] Lokesh Tiwari Jacob M Puliyel. Vitamin D level in slum children of Delhi. Indian Paediatrics. (2004) 41:1076–7. Available online at: https://www.indianpediatrics.net/oct2004/oct-1076-1077.htm 15523147

[57] Marwaha RK Sreenivas V Talwar D Yenamandra VK Challa A Lakshmy R. Impact of solar ultraviolet B radiation (290-320 nm) on vitamin D synthesis in children with type IV and V skin. Br J Dermatol. (2015) 173:604–6. 10.1111/bjd.13887 25939893

[58] Goswami R Gupta N Goswami D Marwaha RK Tandon N Kochupillai N Prevalence and significance of low 25-hydroxy D concentrations in healthy subjects in Delhi. Am J Clin Nutr. (2000) 72:472–5. 10.1093/ajcn/72.2.472 10919943

[59] Puri S Marwaha RK Agarwal N Tandon N Agarwal R Grewal K. Vitamin D status of apparently healthy schoolgirls from two different socioeconomic strata in Delhi: relation to nutrition and lifestyle. Br J Nutr. (2008) 99:876–2. 10.1017/S0007114507831758 17903343

[60] Farrar MD Webb AR Kift R Durkin MT Allan D Herbert A. Efficacy of a dose range of simulated sunlight exposures in raising vitamin D status in South Asian adults: implications for targeted guidance on sun exposure. Am J Clin Nutr. (2013) 97:1210–6. 10.3945/ajcn.112.052639 23615828

[61] Ekbote VH Khadilkar AV Mughal MZ Hanumante N Sanwalka N Khadilkar VV. Sunlight exposure and development of rickets in Indian toddlers. Indian J Pediatr. (2010) 77:61–5. 10.1007/s12098-009-0263-2 19936652

[62] Ritu G Gupta A Vitamin D deficiency in India: prevalence, causalities and interventions. Nutrients. (2014) 6:729–. 10.3390/nu6020729 24566435 PMC3942730

[63] Ritu G Gupta A Fortification of foods with vitamin D in India. Nutrients. (2014) 6:3601–23. 10.3390/nu6093601 25221975 PMC4179178

[64] Ritu G Gupta A Fortification of foods with vitamin D in Indian: strategies targeted at children. J Amer Coll Nut. (2015) 34:263–72. 10.1080/07315724.2014.924450 25790322

[65] Babu SU Calvo MS. Modern India and the vitamin D dilemma: evidence for the need of national food fortification program. Mol Nut Food Res. (2010) 54:1131–47. 10.1002/mnfr.200900480 20440690

[66] Holick MF The global D-Lemma: the vitamin D deficiency pandemic even in sun-drenched countries. J Clin Sci Res. (2018) 7:101–5. 10.4103/JCSR.JCSR_3_19

[67] Pilz S März W Kevin D. Rationale and plan for vitamin D food fortification: a review and guidance paper. Front. Endocrinol. (2018) 9:373. 10.3389/fendo.2018.00373 30065699 PMC6056629

[68] Merewood A Mehta SD Chen TC Bauchner H Holick MF Association between vitamin D deficiency and primary caesarean section. J Clin Endocrinol Metab. (2009) 94:940–5. 10.1210/jc.2008-1217 19106272 PMC2681281

[69] Hubeish M Al Husari H Itani S El Tal R Tamim H Abou Saleh S Maternal vitamin D level and rate of primary Cesarean section. J Clin Gynecol Obstetrics. (2018) 7:43–51. 10.14740/jcgo473w

[70] Sirohi A Pundhira Ghosh S Food fortification: a nutritional management strategy in India. Innovare J Food Sci. (2018) 6:1–8. Available online at: https://www.researchgate.net/profile/Aditya_Pundhir/publication/328723532

[71] Olson CM. Behavioral nutrition interventions using e- and m-health communication technologies: a narrative review. Annu Rev Nutr. (2016) 36:647–64. 10.1146/annurev-nutr-071715-050815 27022772

[72] Scherr RE Laugero KD Graham DJ Cunningham BT Jahns L Lora KR Innovative techniques for evaluating behavioural nutrition interventions. Adv Nutr. (2017) 8:113–25. 10.3945/an.116.013862 28096132 PMC5227983

[73] Rosi A Dall'Asta M Brighenti F Del Rio D Volta E Baroni I. The use of new technologies for nutritional education in primary schools: a pilot study. Public Health. (2016) 140:50–5. 10.1016/j.puhe.2016.08.021 27756495

[74] Goodman S Morrongiello B Randall Simpson J Meckling K. Vitamin D intake among young Canadian adults: validation of a mobile vitamin D calculator app. J Nutr Educ Behav. (2015) 47:242–7. 10.1016/j.jneb.2014.11.006 25959447

[75] Dias D Paulo Silva Cunha J. Wearable health devices-vital sign monitoring, systems and technologies. Sensors. (2018) 18:E2414. 10.3390/s18082414 30044415 PMC6111409

[76] Lee S Oncescu V Mancuso M Mehta S Erickson D. A smartphone platform for the quantification of vitamin D levels. Lab Chip. (2014) 14:1437–42. 10.1039/C3LC51375K 24569647

[77] Harinarayan CV Vitamin D deficiency in sun drenched India – can D-lightful sunlight be a respite? – sunlight D lemma. Proc Indian Natl Sci Acad. (2018) 84:923–35. 10.16943/ptinsa/2018/49447

[78] Harinarayan CV How to treat Vitamin D deficiency in sun-drenched India - guidelines. J Clin Sci Res. (2018) 7:131–40. 10.4103/JCSR.JCSR_1_19

